# Antipsychotic drugs at 75: the past, present, and future of psychosis management

**DOI:** 10.1093/bmb/ldaf016

**Published:** 2025-10-06

**Authors:** Monty Lyman, Robert A McCutcheon

**Affiliations:** Department of Psychiatry, University of Oxford, Warneford Hospital, Warneford Ln, Headington, Oxford OX3 7JX, United Kingdom; Department of Psychiatry, University of Oxford, Warneford Hospital, Warneford Ln, Headington, Oxford OX3 7JX, United Kingdom; Oxford Health NHS Foundation Trust, Warneford Hospital, Warneford Ln, Headington, Oxford OX3 7JX, United Kingdom; Department of Psychosis Studies, Institute of Psychiatry, Psychology and Neuroscience, King’s College London, De Crespigny Park, London, SE5 8AF, United Kingdom

**Keywords:** antipsychotics, psychosis, schizophrenia, precision psychiatry, dopamine

## Abstract

**Introduction:**

The discovery of chlorpromazine in 1950 marked a turning point in psychiatry, and, for the first time, effective pharmacological treatments for psychosis became widely used. Over the following decades, antipsychotics became the cornerstone of schizophrenia treatment, yet their fundamental mechanism—dopamine D2 receptor antagonism—has remained largely unchanged. Now, 75 years on, novel drug classes and advances in mechanistic understanding are reshaping the field.

**Sources of data:**

This review synthesizes findings from clinical trials, neurobiological research, and pharmacological studies, highlighting the evolution of antipsychotic treatment.

**Areas of agreement:**

Antipsychotics reduce positive symptoms, but their efficacy for negative and cognitive symptoms is limited. Clozapine remains the gold standard for treatment-resistant schizophrenia.

**Areas of controversy:**

The typical/atypical distinction is increasingly seen as outdated. The dopamine hypothesis, while central, does not fully explain schizophrenia.

**Growing points:**

Emerging nondopaminergic treatments—such as the muscarinic agonist xanomeline-trospium—offer new therapeutic avenues.

**Areas timely for developing research:**

Further research is needed to determine the clinical utility of nondopaminergic drugs, refine stratified treatment approaches, and integrate precision psychiatry into psychosis management.

## Introduction

Few medical discoveries have reshaped healthcare as profoundly as antipsychotics. Before 1950, the treatment of chronic psychotic disorders such as schizophrenia was ineffective and often brutal. The discovery of chlorpromazine offered—for the first time—a drug that could treat psychosis. Within a few years, pharmacological interventions were a core aspect of schizophrenia treatment, and psychopharmacology became a defining feature of psychiatry more broadly. The discovery of antidepressants in the following years gave the impression that a new era of unstoppable progress had arrived. However, this promise was not fully realized. Over subsequent decades there were minimal advances in terms of antipsychotic mechanism of action and, except for clozapine, only minor improvements in efficacy.

As we approach the 75th anniversary of the discovery of antipsychotics, however, the field is shifting once again. New antipsychotics with entirely novel mechanisms offer hope of both greater efficacy and fewer side effects, while our understanding of current treatments and their optimal use has evolved. The next era of psychosis treatment may be the most transformative yet.

## The pre-antipsychotic age: psychosis before 1950

*‘We are almost forced to drive the devil out of our patients with Beelzebub.’*

*Ladislas Meduna, developer of Cardiazol shock therapy* [1].

For most of human history, psychosis and chronic psychotic disorders were seen through mystical or moralistic lenses. The turn of the 20th century saw them rapidly settle within a medical framework. Emil Kraepelin’s efforts to distil the heterogeneity of his psychiatric presentations led him to distinguish ‘dementia praecox’ from manic-depressive illness in 1893. Subsequently, in 1908, Eugen Bleuler redefined the former as schizophrenia—its Greek roots meaning ‘split mind’, reflecting the fragmentation of thought, perception, and personality [2].

The gold-standard treatment at the time remained long-term asylum confinement. As asylums became overcrowded and under-resourced, the search for biological treatments gained urgency. One of the first major approaches was insulin coma therapy, introduced in the 1920s by Austrian psychiatrist Manfred Sakel [3]. Patients were injected with large doses of insulin, driving them into a hypoglycaemic coma, with the idea that it might ‘reset’ the brain. While some patients showed improvement, the procedure was unpredictable and often led to brain damage or even death.

In the 1930s, convulsive therapies gained traction. Inspired by the erroneous observation that epilepsy and schizophrenia appeared to rarely coexist, Hungarian physician Ladislas Meduna injected high doses of a central and respiratory stimulant—commonly called Cardiazol– into patients to induce seizures [4]. Italian psychiatrists Ugo Cerletti and Lucio Bini refined this approach by developing electroconvulsive therapy (ECT), which induced seizures through electric currents rather than drugs. ECT proved effective in treating severe mental illness and remains in use today.

Psychosurgery, particularly the lobotomy, also became widespread. First performed by the Portuguese neurologist Antonio Moniz in 1935, the procedure involved severing connections of the frontal lobes to reduce agitation [5]. The effect was often catastrophic—patients often became apathetic and severely impaired—but the procedure spread rapidly, with thousands performed worldwide.

By the end of the 1940s, the need for effective treatments for psychotic disorders such as schizophrenia was clear. Existing treatments were crude, high-risk, and often ineffective, driven by the desperation of patients, clinicians, and families. While pharmacological treatments were employed, these were solely sedatives, easing the management of aggression and agitation but having no real impact on the core symptoms of psychosis.

## Barking up the right tree: the serendipitous discovery of antipsychotics

*‘It provokes not any loss in consciousness, not any change in the patient’s mentality but a slight tendency to sleep and above all “disinterest’ for all that goes on around him….These facts let us forsee certain indications for this drug in psychiatry’* [6].

*Laborit, Huguenard & Allaume, A new autonomic stabiliser 4560 RP, February 13 1952.*

For centuries, the only effective treatment for malaria was quinine: a medication derived from the bark of the South American ‘cinchona’ tree. However, wartime shortages of cinchona bark during the First and Second World Wars triggered a rush to produce a synthetic antimalarial drug. In the early 1940s, French chemist Paul Charpentier and his team at Rhone-Poulenc Laboratories began synthesizing phenothiazine derivatives—compounds originally used in the German dye industry that also exhibited antiparasitic properties. Although this search for a malaria treatment ultimately failed, some of these synthetic compounds proved to be effective antihistamines—particularly promethazine [7].

Promethazine—an antagonist of both histamine and muscarinic acetylcholine receptors—had pronounced sedative effects alongside its antihistaminergic properties. Henri-Marie Laborit, a French navy surgeon stationed in North Africa, found the drug to be a powerful addition to his experimental ‘lytic cocktail’: a concoction designed to treat the shock, stress, and anxiety during and after surgery [8]. As part of Laborit’s search for ever more potent compounds, he came across the compound 4560-RP, one of the Rhone-Poulenc derivatives of promazine [9]. When Laborit incorporated this compound into his tranquilizing cocktail, he noticed an unprecedented effect: relaxation and emotional detachment without an impairment of consciousness.

News of chlorpromazine’s effects soon reached psychiatrists Jean Delay and Pierre Deniker at Sainte-Anne Hospital in Paris, who conducted seminal clinical trials of the isolated compound in psychiatric patients [10]. Their first case study was that of a 57-year-old labourer whose manic, erratic behaviour had led him to randomly assault strangers. After 3 weeks of treatment with chlorpromazine, he made a full recovery—one of many such cases [11]. Chlorpromazine became the first ‘neuroleptic’ (Delay and Deniker’s term, meaning ‘taking hold of the nerves’), marking the birth of modern psychopharmacology.

## Discovering dopamine

*‘I felt that a look at the catecholamines might be worthwhile.’*

*Arvid Carlsson, Nobel Prize Lecture, December 8th, 2000.*

While the shortage of cinchona bark indirectly led to the discovery of chlorpromazine, scientists in the early 1950s were also examining the roots of *Rauvolfia serpentina* (Indian snakeroot), a plant long used in Ayurvedic medicine for its tranquilizing properties. In 1952, scientists isolated reserpine, one of its active compounds, which was isolated in 1952 and marketed as an antipsychotic 2 years later [12]. However, while reserpine’s side effects limited its psychiatric use, it became a useful tool for neuroscientists seeking to understand the chemistry of the brain.

In the late 1950s, Swedish pharmacologist Arvid Carlsson discovered that one of reserpine’s most striking side effects—stiffness and slowed movement—could be reversed by L-dopa, the precursor of dopamine. This led to the discovery of dopamine as a neurotransmitter, paving the way for major breakthroughs in neurobiology.

By the mid-1970s, *in vitro* studies had confirmed that the clinical effectiveness of antipsychotics in schizophrenia was strongly linked to their affinity for dopamine D2 receptors [13,14]. Subsequent *in vivo* imaging studies in patients demonstrated that a therapeutic window for D2 occupancy existed between 60% and 80%; any lower and the drug is likely to be ineffective, any higher and the risk of extrapyramidal side effects (EPSEs) such as parkinsonism, and hyperprolactinaemia increases significantly [15].

In addition to the antipsychotic effects of reducing dopaminergic signalling, a large body of research implicates dopamine dysregulation in the pathophysiology of schizophrenia. This is most evident in positron emission tomography studies that have demonstrated elevated presynaptic dopamine synthesis and release capacity in the striatum in schizophrenia, which correlates with the severity of ‘positive symptoms’ such as hallucinations and delusions. It was initially unclear as to whether it is a pre- or postsynaptic pathology, but it appears that presynaptic dopamine is higher in antipsychotic naïve individuals with psychosis compared to controls, whereas postsynaptic increases are a secondary effect of prior antipsychotic treatment [16].

By the late 20th century, a relatively consistent picture had emerged: that in a significant proportion of patients, the positive symptoms of schizophrenia were driven by a hyperdopaminergic state in the striatum, a dysfunction that could be treated by blocking dopamine D2 receptors.

## Atypicals and partial agonists

*‘That which we call a rose, by any other word would smell as sweet’.*

*William Shakespeare, Romeo and Juliet.*

Another notable antihistamine tested as an antipsychotic in the early 1950s was imipramine. However, rather than alleviating psychosis, it unexpectedly demonstrated potent antidepressant effects. With serendipitous circularity, when the Swiss pharmaceutical company Wander AG began synthesizing new compounds based on imipramine in 1958, their efforts led to the discovery of a truly remarkable antipsychotic: clozapine [17]. Successful clinical trials in the 1960s and early adoption of the new drug in the 1970s were halted in 1975 by the discovery of clozapine-induced agranulocytosis (extremely low levels of white blood cells) in a Finnish cohort, leading to eight deaths [18]. However, following a landmark 1988 study that demonstrated its superiority to chlorpromazine in treatment-resistant schizophrenia, clozapine remains—with stringent haematological monitoring requirements—the treatment of choice in those who have not responded to other antipsychotics [19].

Clozapine was not only set apart from existing antipsychotics by its efficacy; it also appeared to potentially involve a different mechanism of action. Notably, it had a reduced affinity for the D2 receptor compared to other antipsychotics at the time and was found to be clinically efficacious at doses associated with a lower dopamine D2 receptor occupancy than other drugs [20]. Its relatively weak and transient affinity for D2 receptors accompanied a broad receptor profile: a high affinity for serotonin 5-HT2A receptors and potent antagonism at muscarinic M1-M5 and histamine H1 receptors, as well as effects on adrenergic α1 and α2 receptors. Intriguingly, clozapine is unique among antipsychotics in that its active metabolite, *N*­desmethylclozapine, is a full agonist at muscarinic M1 receptors, which is relevant given recent discoveries as to the antipsychotic potential of muscarinic agonists (see below) [21]. This pharmacology underlined its strikingly different side-effect profile: one of a lower risk of movement disorders but increased weight gain and other metabolic changes. The search for similar compounds in the 1990s led to a proliferation of what are still commonly termed ‘atypical’ or ‘second-generation’ antipsychotics, including risperidone, olanzapine, and quetiapine. While their efficacy relative to clozapine’s efficacy in treatment-resistant schizophrenia was not entirely clear [22], most retained their distinctive feature of serotonin 5-HT2A antagonism alongside D2 blockade, which was thought to contribute to a lower risk of EPSEs. Although in retrospect this advantage appears largely a reflection of dosing differences, atypicals became the preferred choice in routine practice due to perceived tolerability, expanded indications, and a growing clinical emphasis on minimising movement-related side effects. Large pragmatic trials were undertaken, aimed at clarifying the relative benefits of the newer drugs over their predecessors. These trials, however, demonstrated that these new compounds were not as distinct as originally proposed, with second-generation drugs (other than clozapine) not demonstrating a clinical benefit over older compounds [23,24]. An objective analysis of both the pharmacology and clinical effects of these compounds provides little support for the idea that there are clear differences between first- and second-generation drugs other than the date of marketing approval [25,26].

The turn of the 21st century, however, saw the emergence of a pharmacologically distinct class of antipsychotics. In the late 1980s, scientists at Otsuka Pharmaceutical in Japan set out to address two major limitations of existing treatments: first, their well-established side effects, including EPSEs and metabolic disturbances, that often limited tolerability and adherence. Second, the fact that while D2 receptor blockade in the striatum effectively reduced positive symptoms such as delusions and hallucinations, it failed to produce clinically meaningful improvements in negative symptoms—deficits in motivation, emotional expression, and social engagement—thought to stem from dopaminergic hypoactivity in the prefrontal cortex. The concept of a dopamine partial agonist had been raised in 1983 by Arvid Carlsson, specifically a drug that could act as an antagonist in hyperdopaminergic areas (e.g. the striatum, implicated in psychosis) while simultaneously stimulating underactive dopamine receptors in hypodopaminergic areas (e.g. the prefrontal cortex, implicated in negative symptoms) [27]. The Otsuka team pursued this approach, seeking a compound that could balance dopamine signalling rather than simply block it outright. Their search led to aripiprazole, a novel D2/D3 partial agonist that was United Stated Food and Drug Administration (FDA)-approved in 2002, becoming the first of what is often termed ‘third-generation antipsychotics’. Clinically, aripiprazole offered a reduction in positive symptoms with fewer EPSEs and metabolic side effects than older antipsychotics [28]. A subsequently developed partial agonist, cariprazine, is the only compound to have convincingly shown a benefit on negative symptoms in a head-to-head trial against an alternative antipsychotic [29].

**Figure 1:**
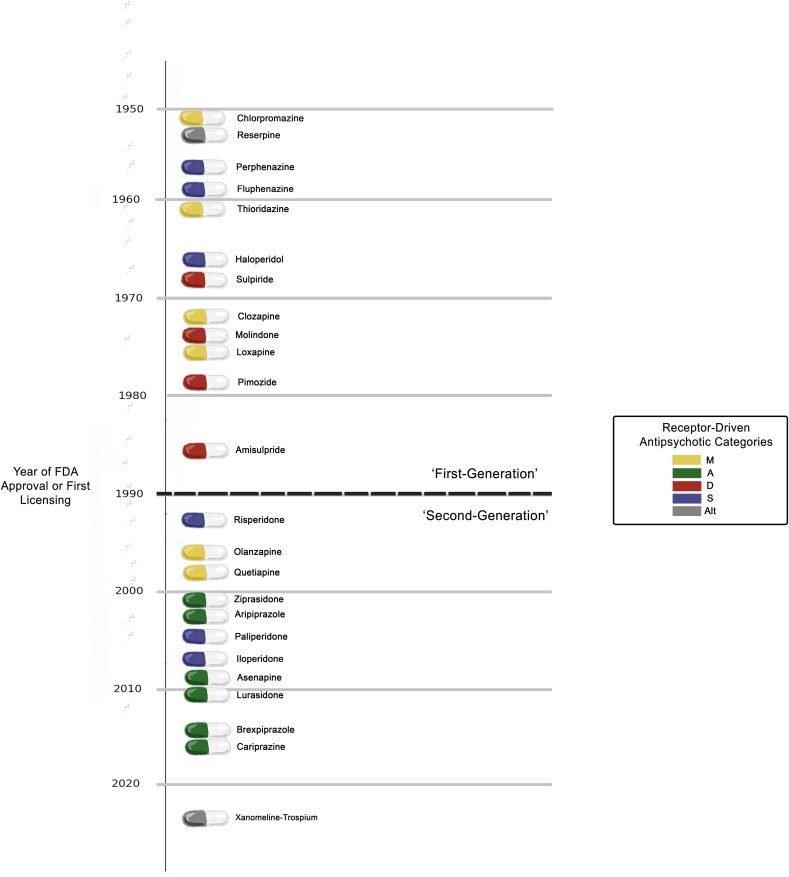
Timeline of antipsychotic drugs. Years indicate when each drug became clinically available, primarily based on FDA approval or first national licencing. Exact dates vary slightly between countries, and some are subject to historical debate depending on how clinical availability is defined. Colours correspond to data-driven receptor binding propensities [26]: M = muscarinic receptor antagonism; A = adrenergic antagonism and dopamine partial agonism; D = dopaminergic antagonism; S = serotonergic and dopaminergic antagonism; Alt = alternative mechanisms. While the distinction between first- and second-generation antipsychotics is increasingly recognized as inadequate, the approximate historical division is often placed around 1990, when clozapine was re-established as the gold standard for treatment-resistant schizophrenia.

Today, clinicians have access to over 25 licenced antipsychotics. While all these drugs, at least in part, exert their effects through the dopamine receptor, each has a unique receptor-binding profile that influences both efficacy and side effects. Despite this pharmacological diversity, the way these drugs are commonly classified has remained largely unchanged for decades, with the traditional typical/atypical distinction increasingly recognized as inadequate for guiding clinical decisions. Attempts have been made to employ a more pharmacologically informed approach to classification, such as the neuroscience-based nomenclature (NbN), which categorizes antipsychotics by their primary pharmacological actions instead of historical or marketing-based distinctions [30]. A more data-driven approach involved the automated analysis of receptor-binding data from over 3000 studies, to identify four distinct pharmacological clusters of antipsychotics based on their receptor affinities [26]. This new system more accurately predicted both efficacy and side-effect burden better than current classifications. The ‘muscarinic antagonism’ group includes drugs with significant M2–M5 muscarinic receptor antagonism, such as clozapine and olanzapine. These agents offer high efficacy but are strongly associated with metabolic side effects. The ‘adrenergic antagonism and dopamine partial agonism’ group consists of newer drugs like aripiprazole, which have slightly lower overall efficacy but fewer side effects. The ‘dopaminergic antagonism’ group—which includes drugs such as amisulpride—is characterized by high efficacy but a greater risk of extrapyramidal side effects and hyperprolactinaemia. The ‘serotonergic and dopaminergic antagonism’ group contains compounds such as risperidone and is associated with a moderate degree of both motor and metabolic side effects. This ‘MADS’ framework (Muscarinic antagonism, Adrenergic antagonism/dopamine partial agonism, Dopaminergic antagonism, Serotonergic/dopaminergic antagonism) is illustrated in Fig. 1.

Recent work has potentially challenged the decades-old axiom that D2 receptor antagonism is key to antipsychotic efficacy [31]. Preclinical work examining the activity of striatal D1 and D2 spiny projection neurons (SPNs) suggested that, despite binding to D2 receptors, the common characteristic of antipsychotic compounds is in fact their ability to modulate the activity of D1-SPNs. Importantly, even novel and experimental antipsychotics with no direct dopamine receptor affinity also achieved this effect, suggesting a broader, circuit-level mechanism of action. The study also found that D1 receptor partial agonists were efficacious through state-dependent modulation, resulting in a stabilization effect: acting as an agonist when dopamine levels were low and an antagonist when dopamine was excessive. Overall, these findings suggest that D1-SPN hyperactivity may play a central role in psychosis and that restoring their balance could be a crucial marker of antipsychotic efficacy.

## Serendipity strikes again: new antipsychotics and novel mechanisms

*‘The skill lies in detecting what actually happened during tests rather than what the theory suggests must have happened.’*

*David Healy, The Creation of Psychopharmacology, 2002.*

The idea that a reduction in cholinergic signalling might contribute to symptoms of Alzheimer’s disease motivated attempts to develop treatments acting via stimulation of cholinergic signalling at muscarinic receptors. In the early 1990s, a newly synthesized muscarinic M1 and M4 receptor agonist called xanomeline appeared to show promise in this endeavour [32]. Initial trials suggested cognitive benefit, but a very curious side effect soon became apparent: a reduction in hallucinations and delusions in patients with psychotic symptoms. Development of xanomeline’s potential as either a treatment for dementia or psychosis, however, was hampered by serious gastrointestinal side effects. Two decades later, a coformulation of xanomeline with trospium chloride—a peripheral muscarinic antagonist that does not cross the blood–brain barrier—ameliorated xanomeline’s side effects without reducing its psychiatric efficacy [33]. Three randomized placebo-controlled trials of xanomeline-trospium have since demonstrated encouraging efficacy in reducing positive symptoms in acute exacerbations of schizophrenia, and it is the first treatment for schizophrenia licensed by the FDA to not possess direct activity at the dopamine receptor [34]. There is also hope for clinically meaningful effects in negative and cognitive symptoms, as well as superior long-term tolerability to existing antipsychotics, which are all being assessed in ongoing clinical trials. The neurobiological and cognitive mechanisms via which muscarinic agonism exerts an antipsychotic effect remain to be established [35]. M4 agonism does appear to attenuate striatal hyperdopaminergia, but the recent failed trials of the pure M4 modulator emraclidine suggests that xanomeline is not just another dopamine modulator and that M1 effects may contribute to efficacy [36].

Alongside other muscarinic agonists, there are a variety of antipsychotics in development that seek to target nondopaminergic mechanisms. A disruption of the signalling between excitatory glutamatergic and inhibitory GABAergic cortical neurons has been proposed to account for cognitive and negative symptoms of the disorder that do not respond to dopamine blockade. Drugs modulating glutaminergic receptors, such as the *N*-methyl-d-aspartate (NMDA) receptor, have therefore been investigated in attempts to restore potential imbalances within these circuits. Iclepertin inhibits glycine transporter 1 (GlyT1), theoretically increasing levels of glycine (a co-agonist of NMDA receptors) in the synaptic cleft and aiding glutamatergic signalling. Despite encouraging improvements on cognitive function in phase II trials, recent phase III results were unsuccessful, in keeping with earlier failed trials of another GlyT1 inhibitor, bitopertin [37]. Similarly, numerous studies of glycine modulatory site agonists, such as glycine and D-serine, have not yet shown convincing clinical effects in human trials [38]. Modulation of the glutaminergic system still holds promise, with evenamide—a voltage-gated sodium channel inhibitor—demonstrating efficacy as an add-on treatment in schizophrenia [39].

While modulation of the NMDA receptor has not yet proven effective in treating idiopathic psychosis, encephalitis secondary to anti-NMDA antibodies can induce a psychotic state. In some of these individuals, complete remission of psychosis has occurred following immunotherapy, with a phase II trial currently exploring the role of immune-directed treatments in those with antibody-associated psychosis [40]. Another unexpected avenue of research concerns cannabidiol (CBD), a nonpsychoactive component of cannabis, which appears to modulate glutaminergic and serotonergic systems indirectly. While CBD is better known for its analgesic, anxiolytic, and anti-inflammatory properties, early clinical trials suggest it may have antipsychotic potential, particularly as an adjunct to existing treatments [41].

While nondopaminergic mechanisms hold significant promise, there remains room for innovation in modulating the dopamine system. While the dopaminergic abnormalities associated with psychotic disorders are primarily presynaptic, current treatments act postsynaptically [42]. Ulotaront, a trace amine-associated receptor 1 (TAAR1) agonist, appears to normalize presynaptic hyperdopaminergia [43]. Despite showing promise in an initial phase two study [44], a subsequent phase three failed, potentially as a result of a remarkably high placebo response [45]. A classical approach may still have benefits; *N*-methylamisulpride—a derivative of amisulpride and potent dopamine D2/3 receptor antagonist—has shown promise in phase II studies and has progressed to phase III trials [46].

## Form matters: the evolving role of long-acting injectables

A final note should be made on the impact of antipsychotic delivery methods, particularly the use of long-acting injectable (LAIs), also known as ‘depots.’ Adherence to antipsychotic medication is notoriously low—resulting from both the adverse effects of the treatments and the limited insight, cognitive impairment, and motivational deficits that characterizes many chronic psychotic illnesses [47]. LAIs are designed to maintain continuous drug exposure over weeks or months, reducing the risk of relapse from missed doses.

First introduced in the 1960s with fluphenazine enanthate and decanoate [48], early LAIs were oil-based formulations injected into muscle, developed to support adherence during the shift to community-based care. Over the past two decades, however, significant innovations in long-term delivery have emerged. In 2003, a risperidone LAI using biodegradable microspheres allowed for steady release via two-weekly intramuscular injections. Since then, a range of delivery technologies—aqueous crystal suspensions, *in situ* depot-forming polymers, and esterified prodrugs—have been developed, all aiming to create sustained-release reservoirs of antipsychotics. Recent advances include longer-interval injections (e.g. 6-month paliperidone, 2-month aripiprazole) and new administration routes (e.g. subcutaneous risperidone). As novel antipsychotics emerge, the parallel development of LAI formulations will be essential—particularly for individuals at greatest risk of relapse, disengagement, or nonadherence. Finally, it is worth noting that other formulations of antipsychotics, which might improve acceptability or adherence—are available, including inhaled loxapine and once weekly oral penfluirdol [49]. Experimental approaches such as intranasal clozapine—designed to reduce systemic side effects through direct-to-brain delivery—are also in development [50].

## Conclusions: looking back and moving forward

The discovery of chlorpromazine in 1950 marked a turning point in psychiatry, transforming psychosis from a condition managed through institutionalization and crude interventions into one that could be treated pharmacologically. In the decades that followed, antipsychotic drugs evolved in terms of their pharmacology, tolerability, and side-effect profiles, but their fundamental mechanism—dopamine D2 receptor antagonism—remained largely unchanged. Now, as we mark the 75th anniversary of antipsychotics, we may be witnessing the dawn of a new era in psychosis treatment.

For the first time, drugs that do not primarily target the dopamine system are reaching clinical practice, challenging long-held assumptions about antipsychotic mechanisms. The recent FDA approval of xanomeline–trospium highlights the potential of muscarinic agonists, and other novel approaches are being actively investigated. At the same time, our understanding of schizophrenia has expanded beyond a simple dopamine imbalance to a more complex, circuit-level disorder, opening new avenues for treatment development. Understanding how these developments should shape treatment approaches is a major challenge [51].

However, despite pharmacological advances, outcomes for individuals with psychotic disorders have not improved as much as might have been hoped. Many patients continue to experience persistent symptoms, severe side effects, and profound social and functional impairments. Reducing the burden of psychosis will require more than just better drugs; it demands a broader approach that integrates early intervention, psychosocial support, and advances in neuroscience to better tailor treatments to individual needs.

Scientific progress in psychiatry has often been driven by a combination of curiosity, technological innovation, and serendipity. The history of antipsychotic discovery—from chlorpromazine’s origins in antihistamines to the unexpected antipsychotic effects of xanomeline—suggests that the future of psychosis treatment may take equally unpredictable turns. If history is any guide, the most transformative discoveries may still lie ahead.

## Data Availability

No new data were generated in support of this review.
